# High-flow nasal cannula improves respiratory impedance evaluated by impulse oscillometry in chronic obstructive pulmonary disease patients: a randomised controlled trial

**DOI:** 10.1038/s41598-022-10873-x

**Published:** 2022-04-28

**Authors:** Yen-Liang Kuo, Chen-Lin Chien, Hsin-Kuo Ko, Hsin-Chih Lai, Tzu-Lung Lin, Li-Na Lee, Chih-Yueh Chang, Hsiang-Shi Shen, Chia-Chen Lu

**Affiliations:** 1 Division of Chest Medicine, Department of Internal Medicine, Fu Jen Catholic University Hospital, Fu Jen Catholic University, New Taipei City, 24352 Taiwan, ROC; 2 School of Medicine, College of Medicine, Fu Jen Catholic University, New Taipei City, 24205 Taiwan, ROC; 3 Master of Science Program in Transdisciplinary Long-Term Care, College of Medicine, Fu Jen Catholic University, New Taipei City, 24205 Taiwan, ROC; 4 Graduate Institute of Biomedical Sciences, College of Medicine, Chang Gung University, Gueishan, 33302 Taoyuan Taiwan, ROC; 5 Division of General Medicine, Department of Internal Medicine, Fu Jen Catholic University Hospital, Fu Jen Catholic University, New Taipei City, 24352 Taiwan, ROC; 6 Department of Respiratory Therapy, College of Medicine, Fu-Jen Catholic University, No. 510, Zhongzheng Rd., Xinzhuang Dist., New Taipei City, 242 Taiwan, ROC; 7 Division of Respiratory Therapy, Department of Chest Medicine, Taipei Veterans General Hospital, Taipei, 112 Taiwan, ROC; 8 School of Medicine, National Yang Ming Chiao Tung University, Taipei, 112 Taiwan, ROC; 9 Department of Medical Biotechnology and Laboratory Science, College of Medicine, Chang Gung University, Gueishan, 33302 Taoyuan Taiwan, ROC; 10 Microbiota Research Center and Emerging Viral Infections Research Center, Chang Gung University, Gueishan, 33302 Taoyuan Taiwan, ROC; 11 Research Center for Chinese Herbal Medicine and Research Center for Food and Cosmetic Safety, College of Human Ecology, Chang Gung University of Science and Technology, Gueishan, 33303 Taoyuan Taiwan, ROC; 12 Department of Laboratory Medicine, Linkou Chang Gung Memorial Hospital, Taoyuan, 33305 Taiwan, ROC; 13 Central Research Laboratory, Xiamen Chang Gung Allergology Consortium, Xiamen Chang Gung Hospital, Xiamen, China; 14 Department of Respiratory Therapy, College of Medicine, Chang Gung University, Gueishan, Taoyuan 33302 Taiwan, ROC

**Keywords:** Physiology, Medical research

## Abstract

Non-pharmacological treatment with high-flow nasal cannula (HFNC) may play a vital role in treatment of patients with chronic obstructive pulmonary disease (COPD). To evaluate the efficacy of HFNC, impulse oscillation system (IOS) is a new noninvasive technique in measuring the impedance of different portions of lungs. It shows higher sensitivity in contrast to conventional pulmonary function tests (PFT). However, whether IOS is an appropriate technique to evaluate the efficacy of HFNC in improving the impedance of small airways or peripheral lung in patients with COPD is still unclear. We enrolled 26 stable COPD participants randomised into two groups receiving HFNC or nasal cannula (NC) for 10 min followed by a 4-week washout period and crossover alternatively. IOS was used to detect the difference of respiratory impedance after HFNC or NC interventions. IOS parameters, PFT results, transcutaneous partial pressure of carbon dioxide, peripheral oxygen saturation, body temperature, respiratory rate, pulse rate, and blood pressure at the time of pre-HFNC, post-HFNC, pre-NC, and post-NC, were collected and analysed using SPSS (version 25.0, IBM, Armonk, NY, USA). The IOS measurement indicated that HFNC significantly improved R5, R5% predicted, R5–R20, X5-predicted, and Fres compared with NC, whereas no significant difference was observed through the PFT measurement. The beneficial effect of HFNC in improving small airway resistance and peripheral lung reactance compared with that of NC in patients with stable COPD was confirmed through IOS measurement.

Trial registration: ClinicalTrials.gov NCT05130112 22/11/2021.

## Introduction

Chronic obstructive pulmonary disease (COPD) has persistent respiratory symptoms and airflow limitation with multiple comorbidities and continues to place a large burden on public health^1–4^. The contribution of small airways in chronic respiratory diseases has received increasing attention. Peripheral airways are the major site of airway obstruction in patients with COPD^4–15^. In patients with COPD, the presence of small airway disease (SAD) assessed using the impulse oscillation system (IOS) progressively increases with Global Initiative for Chronic Obstructive Lung Disease (GOLD) classifications (49%, 88%, 61%, and 96% from GOLD A to D, respectively), and it is closely related to the strong effect of the disease on health status^10^.

The treatment of small airway dysfunction is essential. In addition to common pharmacological inhalation therapies (such as bronchodilator drugs or anti-inflammatory drugs) for small airway dysfunction in COPD^16^, nonpharmacological treatments, such as pulmonary rehabilitation and high-flow nasal cannula (HFNC), may play a vital role in COPD treatment^17,18^. HFNC is an adjuvant support system that can reduce upper airway inspiratory resistance through a strong flow gas of up to 60 L/min^19–26^. HFNC might affect the treatment of patients with stable COPD^27^. However, no definitive guidelines for the use HFNC in patients with COPD have been established. In addition, the efficacy of HFNC for different levels in respiratory impedance is unclear.

The pulmonary function test (PFT) is the most common technique for assessing small airways in terms of forced expiratory flow between 25 and 75% of the forced vital capacity^28,29^, total lung capacity, functional residual capacity, and residual volume by body plethysmography^30^. IOS is a new technique for measuring respiratory impedance (both resistance and reactance). It is used to assess functional abnormalities in the small airway and longitudinally monitor the effects of interventions and pharmacological treatments for chronic lung diseases^31–33^. However, whether IOS is appropriate for detecting impedance changes in small airways or peripheral lung after HFNC treatment remains unclear.

This randomised crossover trial investigated the efficacy of 10-min HFNC and nasal cannula (NC) use in patients with stable COPD by using IOS indicators of respiratory impedance (resistance and reactance), namely R5, R5% predicted, R20, R20% predicted, R5–R20 (the value of R5 difference to the value of R20), X5-predicted (the value of X5 difference to the value of predicted X5), resonant frequency (Fres), and area under reactance curve between 5 Hz and resonant frequency (Ax). Physiological parameters were also assessed. This is the first study to evaluate the efficacy of HFNC in improving respiratory impedance compared with that of NC through IOS. Our results may provide useful reference for clinicians to develop a clinical treatment policy to treat patients with stable COPD.

## Materials and methods

### Participants

**Figure 1:**
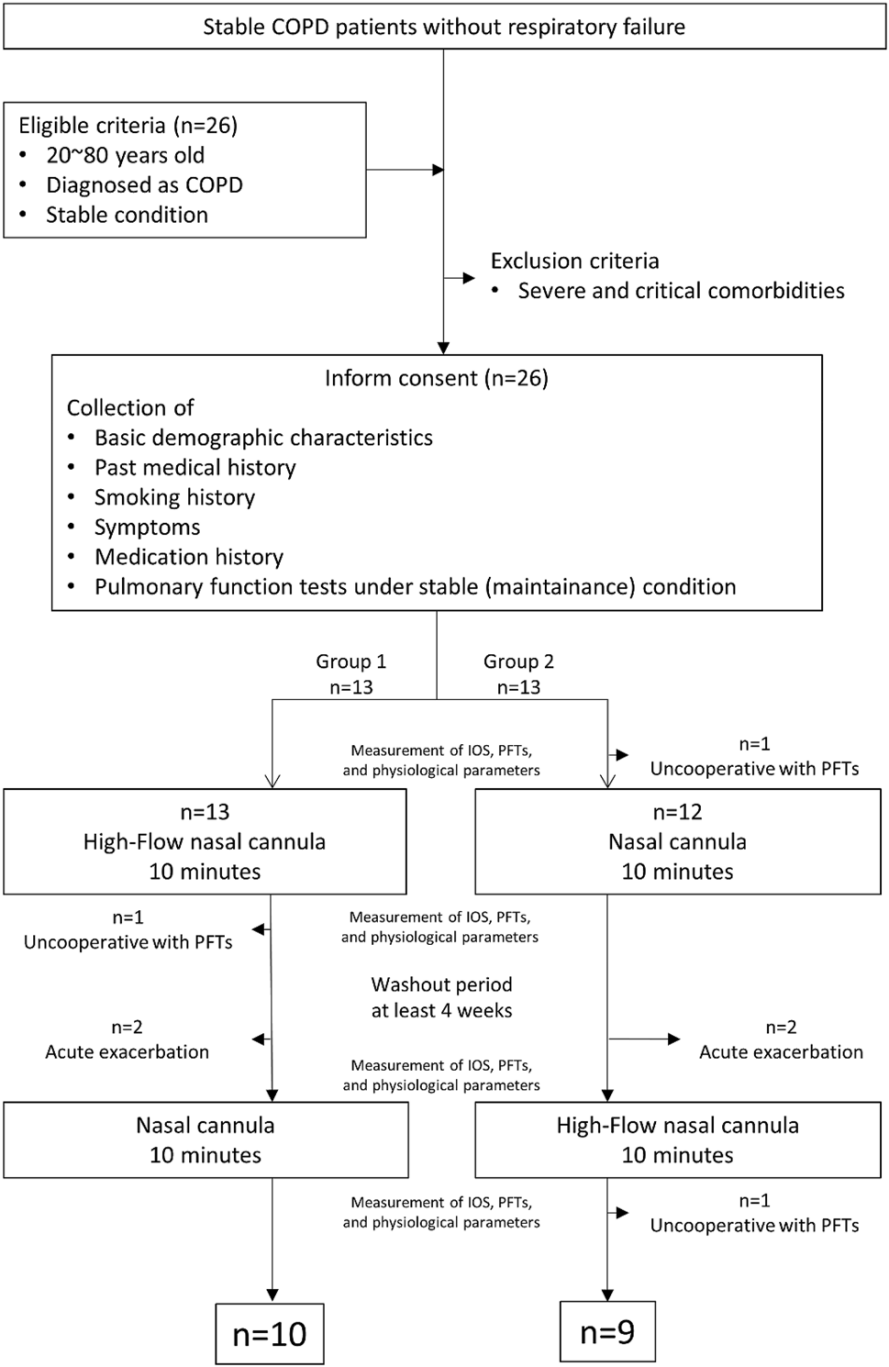
Flowchart of participant selection.

We enrolled 26 participants with stable COPD from the chest medicine outpatient department of Fu-Jen Catholic University Hospital (New Taipei City, Taiwan) between December 2019 and March 2020 (Fig. 1). The inclusion criteria were as follows: (1) age of ≥ 20 years, (2) diagnosis of COPD made by pulmonologist (Kuo YL) if the patient had a long-term smoking history > 10 pack years (20 cigarettes per day for years) or noxious gas exposure at least 10 years (such as incense exposure)^34^, typical COPD clinical manifestations, and airflow limitation with a postbronchodilator FEV1/FVC ratio of < 0.7 in spirometry^4^, and (3) provision of written informed consent. The exclusion criteria were as follows: (1) severe and unstable comorbidities or active malignancy, (2) history of obstructive sleep apnoea syndrome, (3) COPD exacerbation within the 4 weeks prior, (4) current use of long-term oxygen therapy or noninvasive ventilation or use within the 6 weeks prior, (5) cognitive impairment or a psychiatric disorder, and (6) pregnancy.

### Study design

We performed a prospective randomised crossover trial to determine the efficacy of 10-min (by chronograph) HFNC and NC use in patients with stable COPD. Patients who met the inclusion criteria were recruited at participating sites and simple randomly assigned to one of two groups by investigators. Group 1 received HFNC for 10 min (Period 1) and NC for 10 min (Period 2) after a 4-week washout period; Group 2 received NC for 10 min (Period 1) and HFNC for 10 min (Period 2) after a 4-week washout period. In parallel, routine medications for all participants during experimental period were also maintained. All participants received routine inhaler medications between about 8 A.M. to 10 A.M. while the measurements were performed in the afternoon (around 2 P.M. to 4 P.M.) during each visit. We maintained similar evaluation time zone/duration each time for each patient. Immediately after HFNC or NC for 10 min, patients received IOS and PFT. The following baseline data were collected: demographic characteristics, medical history, smoking history, symptoms, medications, and the results of PFTs conducted during stable patient conditions within 1 year prior to enrolment. We performed IOS for 30 s according to the default setting of the machine and collected the parameters first, then performed PFT and collected parameters, and then collected the data with transcutaneous partial pressure of carbon dioxide (TcPCO_2_), saturation of peripheral blood (SpO_2_), body temperature (BT), respiratory rate (RR), pulse rate (PR), and blood pressure (BP) at the time of pre-HFNC, post-HFNC, pre-NC, and post-NC immediately. The primary outcome was change in IOS parameters, including R5, R5% predicted, R20, R20% predicted, R5–R20 (the value of R5 difference to the value of R20), X5-predicted (the value of X5 difference to the value of predicted X5), resonant frequency (Fres), and area under reactance curve between 5 Hz and resonant frequency (Ax). The secondary outcomes were changes in TcPCO_2_, SpO_2_, RR, PR, BP, BT, and PFT parameters.

### HFNC and NC

HFNC (fraction of inspired oxygen [FiO_2_] with approximately 0.22 and gas flow with 50 L/min for 10 min) was administered using the MyAIRVO 2 device (Fisher & Paykel Healthcare, Auckland, New Zealand), which provides humidification and high-flow medical gas through an Optiflow NC interface (Fisher & Paykel Healthcare, Auckland, New Zealand). The investigator was allowed to downtitrate the flow gradually to a minimum of 20 L/min if the participants reported discomfort from HFNC. NC was administered at 1 L/min (FiO_2_: approximately 0.24) for 10 min.

### Measurements

The PFT (Medical Graphics Corporation, Minnesota, USA) was performed by trained operators in accordance with the guidelines of the American Thoracic Society, European Respiratory Society, and operator manual (including pulmonary reference) from https://mgcdiagnostics.com/^35,36^. Predicted values of spirometry and plethysmography were calculated in accordance with GLI 2012 and ITS equation, respectively^37,38^. IOS was also performed by trained operators according to operator manual of Masterscreen IOS (Vyaire Medical, Würzburg, Germany). Results were shown with an average of 3 consecutive technically acceptable tests for every participant (each test with 30 s of tidal breathing measurement (totally within 2 min). Theory technical realisation and reference values were according to Vogel J, Smidt U. Impulse oscillometry: analysis of lung mechanics in general practice and the clinic, epidemiological and experimental research. Frankfurt am Main: Pmi Verlagsgruppe; 1994. SpO_2_ was measured using pulse oximetry. We also recorded TcPCO_2_ using the SenTec Digital Monitor and V-sign Sensor (SenTec, Therwil, Switzerland). All measurements were performed at our hospital. Usual medications were administrated around 8 a.m. to 10 a.m. and we performed the measurements in the afternoon (around 2 p.m. to 4 p.m.) during each visit.

### Statistical analysis

Statistical analysis was performed using SPSS (version 25.0, IBM, Armonk, NY, USA) software. Descriptive data are expressed as means ± standard deviation. Student’s t test was used to compare the continuous variables. The chi-square or Fisher’s exact test was used to compare the categorical variables. An analysis of covariance was performed to evaluate effects of treatment (pretest data as covariate). The models examined the differences in the measurements before and after 10 min of HFNC or NC. All tests were two-sided and performed at a significance level of 0.05. The results were evaluated through an intention-to-treat analysis.

### Sample size

This is the first study to evaluate the efficacy of HFNC in improving respiratory impedance through IOS compared with that of NC, so we were unable to estimate the minimum number of participants. We set the number of participants to be around 20 for statistical analysis.

### Study conduct, approval, and registration

This trial was conducted in accordance with the Declaration of Helsinki and the Ethical Guidelines for Medical and Health Research Involving Human Subjects. The study protocol was approved by the Fu-Jen Catholic University ethics committees (C107177), and written informed consent was obtained from all participants before the trial.

## Results

### Demographic and baseline characteristics of participants

A total of 26 patients with COPD from December 2019 to March 2020 were enrolled (Fig. 1). In Group 1, one patient was excluded because of missing data due to refusal to participate in the PFT after the HFNC treatment, and two were excluded because of acute exacerbation of COPD during the washout period. In Group 2, two patients were excluded because of acute exacerbation of COPD during the washout period, and two were excluded because of missing data due to refusal to participate in the PFT before the NC treatment and after the HFNC treatment. A total of 10 patients in Group 1 and 9 patients in Group 2 completed the trial.

The mean age of the participants was 70.4 ± 8.67 years, and most (94.7%) were men. The mean body mass index (BMI) was 22.9 ± 3.13. The most common symptoms of COPD were sputum (84.2%) and cough (73.7%). The severity of airway obstruction was diagnosed in accordance with GOLD guidelines from stages I to IV: stage I (n = 1), stage II (n = 6), stage III (n = 9), and stage IV (n = 3) (Table 1).

**Table 1:** Basic demographic characteristics of stable COPD patients.

	Total (n = 19)	Group 1 (n = 10) HFNC then NC	Group 2 (n = 9) NC then HFNC	*p *value
Age (year)	70.4 ± 8.7	74.3 ± 5.6	66.0 ± 9.6	0.04*
Male sex	18 (94.7)	10 (100%)	8 (88.9)	0.28
Height (cm)	162.5 ± 6.6	162.3 ± 6.2	162.8 ± 7.3	0.88
Body weight (kg)	60.7 ± 9.9	63.7 ± 7.5	57.4 ± 11.6	0.18
BMI (kg/m^2^)	22.9 ± 3.1	24.3 ± 2.3	21.3 ± 3.3	0.03*
Smoking history (pack per day*years)	55.3 ± 33.6	61.0 ± 31.8	48.9 ± 36.2	0.45
**Symptoms**				
Wheeze	1 (5.3)	0 (0)	1 (11.1)	0.28
Cough	14 (73.7)	7 (70.0)	7 (77.8)	0.70
Sputum	16 (84.2)	9 (90.0)	7 (77.8)	0.47
Dyspnea	6 (31.6)	2 (20.0)	4 (44.4)	0.25
**Medication**				
LAMA + LABA	8 (42.1)	3 (30.0)	5 (55.6)	0.45
LAMA + LABA + ICS	4 (21.1)	2 (20.0)	2 (22.2)	
ICS + LABA	2 (10.5)	2 (20.0)	0 (0)	
LAMA	5 (26.3)	3 (30.0)	2 (22.2)	
Pulmonary rehabilitation	4 (21.1)	2 (20.0)	2 (22.2)	0.91
**Comorbidity**				
Coronary artery disease	3 (15.8)	1 (10.0)	2 (22.2)	0.47
Chronic heart failure	1 (5.3)	1 (10.0)	0 (0)	0.33
Old stroke	0 (0)	0 (0)	0 (0)	N/A
**COPD severity**				
GOLD 1 (FEV1 ≥ 80% predicted)	1 (5.3)	1 (10.0)	0 (0)	0.71
GOLD 2 (50% ≤ FEV1 ≤ 79% predicted)	6 (31.6)	3 (30.0)	3 (33.3)	
GOLD 3 (30% ≤ FEV1 ≤ 49% predicted)	9 (47.4)	5 (50.0)	4 (44.4)	
GOLD 4 (FEV1 < 30% predicted)	3 (15.8)	1 (10.0)	2 (22.2)	
**Spirometry**				
FEV1/FVC (%)	45.8 ± 10.2	49.5 ± 11.4	41.7 ± 7.3	0.10
FEV1 (L)	1.00 ± 0.4	1.1 ± 0.4	0.9 ± 0.3	0.48
FEV1 (% predicted)	45.5 ± 15.6	49.8 ± 17.4	40.8 ± 12.6	0.22
FVC (L)	2.2 ± 0.7	2.2 ± 0.6	2.3 ± 0.8	0.76
FVC (% predicted)	72.6 ± 18.5	74.3 ± 18.9	70.8 ± 18.9	0.69
FEV3 (L)	1.6 ± 0.5	1.6 ± 0.5	1.5 ± 0.5	0.74
FEV3 (% predicted)	53.0 ± 13.7	56.0 ± 13.9	49.6 ± 13.4	0.32
FEF 25–75% (L/s)	0.4 ± 0.2	0.5 ± 0.3	0.3 ± 0.1	0.22
FEF 25–75% (% predicted)	26.6 ± 19.9	34.4 ± 24.2	17.9 ± 8.5	0.70
PEF (L/s)	2.6 ± 1.0	2.9 ± 1.0	2.3 ± 0.9	0.21
PEF (% predicted)	43.8 ± 17.6	51.3 ± 18.3	35.6 ± 13.1	0.05*
**Body plethysmography**				
RV (L)	3.7 ± 2.1	3.8 ± 2.8	3.6 ± 1.0	0.86
RV (% predicted)	172.8 ± 93.6	169.6 ± 122.3	176.3 ± 53.3	0.88
TLC (L)	6.1 ± 2.1	6.1 ± 2.6	6.0 ± 1.3	0.94
TLC (% predicted)	106.6 ± 33.9	109.5 ± 44.9	103.3 ± 16.9	0.70
RV/TLC (%)	59.3 ± 11.4	58.3 ± 13.6	60.4 ± 9.0	0.69
FRC (L)	4.6 ± 2.0	4.8 ± 2.7	4.4 ± 1.0	0.71
FRC (% predicted)	151.2 ± 64.5	154.9 ± 86.3	147.0 ± 30.9	0.80
IC (L)	1.5 ± 0.4	1.3 ± 0.3	1.6 ± 0.5	0.15
IC (% predicted)	57.1 ± 22.4	58.4 ± 28.8	55.7 ± 13.8	0.80
Small airway disease (FEF 25–75% < 65% predicted)	18 (94.7)	9 (90.0)	9 (100.0)	0.33

Continuous data are expressed as mean ± SD with t test.

Categorical data are expressed as number (%) with Chi-square test.

*COPD* chronic obstructive pulmonary disease, *HFNC* high flow nasal cannula, *NC* nasal cannula, *BMI* body mass index, *LAMA* long-acting muscarinic antagonists, *LABA* long-acting β2 sympathomimetic agonists, *ICS* inhaled corticosteroid, *N/A* not applicable, *GOLD* global initiative for chronic obstructive lung disease, *FEV1* forced expiratory volume that has been exhaled at the end of the first second of forced expiration, *FVC* forced vital capacity, *FEV3* forced expiratory volume that has been exhaled at the end of the third second of forced expiration, *FEF 25–75%* forced expiratory flow at 25–75% of the pulmonary volume, *PEF* peak expiratory flow, *RV* residual volume, *TLC* total lung capacity, *FRC* functional residual capacity, *IC* inspiratory capacity.

*Significance between group 1 and group 2.

Table 1 presents the participants’ clinicodemographic. Baseline PFT results within 1 year prior to enrolment were collected during stable patient conditions and regular maintenance inhalation therapy with holding the dose of that day (stable trough PFT). No significant between-group differences were observed in the baseline spirometry or body plethysmography parameters. All patients had a decreased forced expiratory flow at 25–75% of the pulmonary volume (FEF 25–75%) of < 65% predicted, and the mean residual lung volume (RV) (L) was 3.72 ± 2.08, with a mean RV (% predicted) of 172.8 ± 93.6, indicating small airway disease (dysfunction).

### HFNC significantly reduces small airway resistance and peripheral lung reactance

Table 2 presents the IOS measurements of the participants. Before and after the HFNC and NC treatments, the mean R5 (kPa/[L/s]) was 0.58 ± 0.17, 0.54 ± 0.12, 0.55 ± 0.16, and 0.58 ± 0.15, respectively; mean R5 (% predicted) was 182.37 ± 55.57, 168.84 ± 42.29, 169.11 ± 41.42, and 179.47 ± 44.74, respectively; mean R20 (kPa/[L/s]) was 0.33 ± 0.64, 0.31 ± 0.50, 0.33 ± 0.58, and 0.32 ± 0.60, respectively; mean R20 (% predicted) was 119.00 ± 25.44, 113.26 ± 21.22, 117.84 ± 22.04, and 116.42 ± 21.84, respectively; mean R5–R20 (kPa/[L/s]) was 0.25 ± 0.12, 0.22 ± 0.10, 0.22 ± 0.13, and 0.25 ± 0.11, respectively; mean X5-predicted (kPa/[L/s]) was − 0.30 ± 0.16, − 0.24 ± 0.17, − 0.28 ± 0.16, and − 0.31 ± 0.16, respectively; mean Fres (Hz) was 25.03 ± 4.94, 23.94 ± 5.76, 22.84 ± 5.51, and 25.14 ± 5.92, respectively; and mean Ax (kPa/L) was 2.93 ± 1.44, 2.66 ± 1.41, 2.59 ± 1.59, and 2.99 ± 1.68, respectively.

**Table 2:** The effect of HFNC and NC intervention on respiratory impedance evaluated by IOS.

	HFNC (n = 19)	Mean difference	NC (n = 19)	Mean difference	*p* value
**R5 [kPa/ (L/s)]**					
Before	0.58 ± 0.17	−0.04	0.55 ± 0.16	+ 0.03	0.04*
After	0.54 ± 0.12		0.58 ± 0.15		
**R5 (% predicted)**					
Before	182.37 ± 55.57	−13.53	169.11 ± 41.42	+ 10.36	0.05*
After	168.84 ± 42.29		179.47 ± 44.74		
**R20 [kPa/ (L/s)]**					
Before	0.33 ± 0.64	−0.02	0.33 ± 0.58	−0.01	0.22
After	0.31 ± 0.50		0.32 ± 0.60		
**R20 (% predicted)**					
Before	119.00 ± 25.44	−5.74	117.84 ± 22.04	−1.42	0.26
After	113.26 ± 21.22		116.42 ± 21.84		
**R5–R20 [kPa/ (L/s)]**					
Before	0.25 ± 0.12	−0.03	0.22 ± 0.13	+ 0.03	0.04*
After	0.22 ± 0.10		0.25 ± 0.11		
**X5-predicted [kPa/ (L/s)]**					
Before	−0.30 ± 0.16	0.06	−0.28 ± 0.16	−0.03	0.03*
After	−0.24 ± 0.17		−0.31 ± 0.16		
**Fres (Hz)**					
Before	25.03 ± 4.94	−1.09	22.84 ± 5.51	+ 2.30	0.01*
After	23.94 ± 5.76		25.14 ± 5.92		
**Ax (kPa/L)**					
Before	2.93 ± 1.44	−0.27	2.59 ± 1.59	+ 0.40	0.09
After	2.66 ± 1.41		2.99 ± 1.68		

Analysis of Covariance (ANCOVA) test, p < 0.05.

*HFNC* high flow nasal cannula, *NC* nasal cannula, *IOS *impulse oscillometry, *R5* resistance at 5 Hz, *R20* resistance at 20 Hz, *R5 − R20*  difference between R5 and R20, *X5* reactance at 5 Hz, *Fres* resonant frequency, *Ax* area under reactance curve between 5 Hz and resonant frequency.

*Significance between high-flow nasal cannula and nasal cannula.

**Figure 2:**
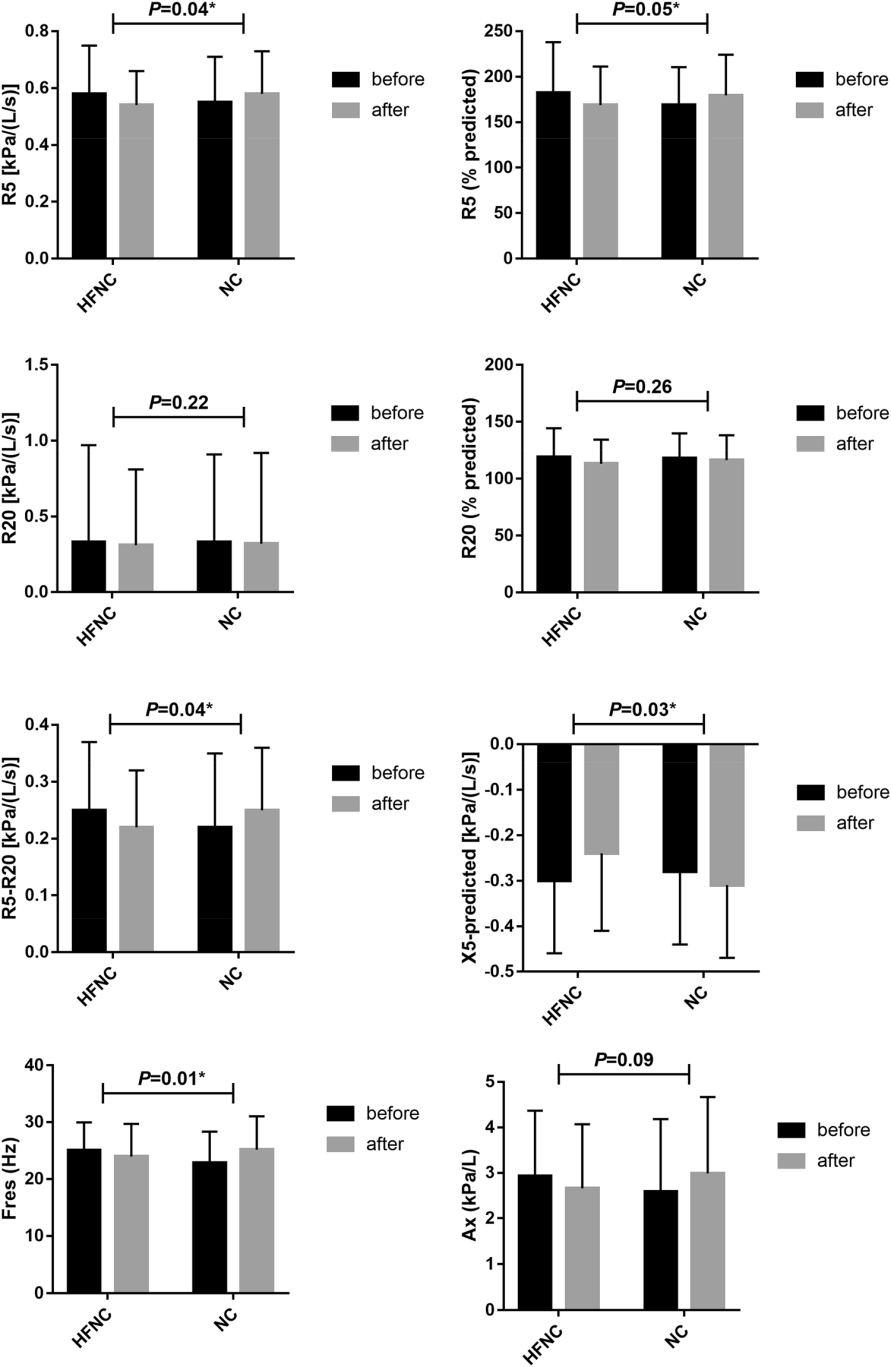
The high-flow nasal cannula (HFNC) intervention reduced total airway resistance, small airway resistance, and reactance in patients with stable COPD. The HFNC intervention decreased resistance (R)5 (kPa/[L/s]) (*p* = 0.04), R5 (% predicted) (*p* = 0.05), R5–R20 (kPa/[L/s]) (*p* = 0.04), X5-predicted (kPa/[L/s]) (*p* = 0.03), and Fres (Hz) (*p* = 0.01), indicating that HFNC improves small airway resistance and peripheral lung reactance.

We performed Wilcoxon signed rank analysis and there is no significant difference of R5, R5%, R20, R20%, R5-20, X5-predicted, nor Ax after NC alone for 10 min. Using Wilcoxon signed rank analysis, there are significant improvement of R5, R5%, R20, R20%, and X5-predicted after HFNC alone for 10 min. Then we used ANCOVA test. Compared with the NC intervention, the HFNC intervention significantly decreased R5 (kPa/[L/s]) (*p* = 0.04), R5 (% predicted) (*p* = 0.05), R5–R20 (kPa/[L/s]) (*p* = 0.04), X5-predicted (kPa/[L/s]) (*p* = 0.03), and Fres (Hz) (*p* = 0.01) (Fig. 2). No significant change was observed in R20 (kPa/[L/s]) (*p* = 0.22), R20 (% predicted) (*p* = 0.26), and Ax (kPa/L) (*p* = 0.09) between the HFNC and NC interventions.

### Spirometry and body plethysmography parameters

Table 3 presents the spirometry and body plethysmography measurements of the participants. No significant change in spirometry parameters (forced expiratory volume in 1 s [FEV1]/forced vital capacity [FVC] [%]: *p* = 0.72; FEV1 [L]: *p* = 0.31; FEV1 [% predicted]: *p* = 0.24; FVC [L]: *p* = 0.32; FVC [% predicted]: *p* = 0.37; FEV3 [L]: *p* = 0.36; FEV3 [% predicted]: *p* = 0.36; FEF 25–75% [L/s]: *p* = 0.27; FEF 25–75% [% predicted]: *p* = 0.17; peak expiratory flow [PEF] [L/s]: *p* = 0.66; PEF [% predicted]: *p* = 0.67) or in body plethysmography parameters (RV [L]: *p* = 0.73; RV [% predicted]: *p* = 0.71; TLC [L]: *p* = 0.79; total lung capacity [TLC] [% predicted]: *p* = 0.55; RV/TLC [%]: *p* = 0.87; functional residual capacity [FRC] [L]: *p* = 0.56; FRC [% predicted]: *p* = 0.48; inspiratory capacity [IC] [L]: *p* = 0.37; and IC [% predicted]: *p* = 0.38) was observed between the HFNC and NC interventions.

**Table 3:** The effect of HFNC and NC intervention on respiratory impedance evaluated by PFT.

	HFNC (n = 19)	Mean difference	NC (n = 19)	Mean difference	*p* value
**FEV1/FVC (%)**					
Before	45.68 ± 12.16	−0.05	46.32 ± 11.72	+ 0.26	0.72
After	45.63 ± 11.44		46.58 ± 12.31		
**FEV1 (L)**					
Before	1.01 ± 0.33	+ 0.02	1.01 ± 0.38	+ 0.04	0.31
After	1.03 ± 0.32		1.05 ± 0.36		
**FEV1 (% predicted)**					
Before	47.53 ± 16.68	+ 0.63	47.42 ± 18.71	+ 1.79	0.24
After	48.16 ± 16.22		49.21 ± 18.58		
**FVC (L)**					
Before	2.27 ± 0.61	+ 0.03	2.21 ± 0.61	+ 0.08	0.32
After	2.30 ± 0.60		2.29 ± 0.61		
**FVC (% predicted)**					
Before	74.58 ± 14.70	+ 1.05	72.79 ± 16.76	+ 2.47	0.37
After	75.63 ± 14.42		75.26 ± 15.44		
**FEV3 (L)**					
Before	1.60 ± 0.45	+ 0.02	1.59 ± 0.51	+ 0.05	0.36
After	1.62 ± 0.44		1.64 ± 0.45		
**FEV3 (% predicted)**					
Before	55.05 ± 14.53	+ 0.48	54.68 ± 16.72	+ 1.43	0.36
After	55.53 ± 14.27		56.11 ± 15.20		
**FEF 25–75% (L/s)**					
Before	0.42 ± 0.23	−0.01	0.42 ± 0.26	+ 0.02	0.27
After	0.41 ± 0.22		0.44 ± 0.26		
**FEF 25–75% (% predicted)**					
Before	28.05 ± 18.68	−0.37	27.89 ± 18.76	+ 1.74	0.17
After	27.68 ± 18.32		29.63 ± 22.06		
**PEF (L/s)**					
Before	2.32 ± 0.73	+ 0.05	2.46 ± 0.86	-0.02	0.66
After	2.37 ± 0.80		2.44 ± 0.82		
**PEF (% predicted)**					
Before	38.37 ± 11.67	+ 1.00	40.95 ± 15.33	0	0.67
After	39.37 ± 13.07		40.95 ± 15.84		
**RV (L)**					
Before	3.42 ± 0.82	+ 0.25	3.42 ± 0.77	+ 0.12	0.73
After	3.67 ± 1.31		3.54 ± 0.96		
**RV (% predicted)**					
Before	160.3 ± 44.2	+ 12.5	160.0 ± 40.8	+ 6.10	0.71
After	172.8 ± 69.3		166.1 ± 49.8		
**TLC (L)**					
Before	5.80 ± 1.02	+ 0.26	5.79 ± 0.88	+ 0.18	0.79
After	6.06 ± 1.17		5.97 ± 1.03		
**TLC (% predicted)**					
Before	119.9 ± 26.6	+ 8.50	118.1 ± 17.8	+ 3.10	0.55
After	128.4 ± 47.8		121.2 ± 16.7		
**RV/TLC (%)**					
Before	58.9 ± 8.90	+ 0.80	58.7 ± 8.4	+ 0.40	0.87
After	59.7 ± 11.2		59.1 ± 10.2		
**FRC (L)**					
Before	4.45 ± 0.89	+ 0.26	4.44 ± 0.88	+ 0.09	0.56
After	4.71 ± 1.18		4.53 ± 0.93		
**FRC (% predicted)**					
Before	147.4 ± 29.8	+ 12.1	147.5 ± 33.7	+ 3.10	0.48
After	159.5 ± 57.9		150.6 ± 32.8		
**IC (L)**					
Before	1.34 ± 0.40	0	1.36 ± 0.34	+ 0.08	0.37
After	1.34 ± 0.50		1.44 ± 0.38		
**IC (% predicted)**					
Before	75.4 ± 23.9	−0.10	77.3 ± 28.7	+ 5.10	0.38
After	75.3 ± 29.7		82.4 ± 33.7		

Analysis of Covariance (ANCOVA) test, p < 0.05.

*HFNC* high flow nasal cannula, *NC* nasal cannula, *FEV1* forced expiratory volume that has been exhaled at the end of the first second of forced expiration, *FVC* forced vital capacity, *FEV3* forced expiratory volume that has been exhaled at the end of the third second of forced expiration, *FEF 25–75%* forced expiratory flow at 25–75% of the pulmonary volume, *PEF* peak expiratory flow, *RV* residual volume, *TLC* total lung capacity, *FRC* functional residual capacity, *IC*  inspiratory capacity.

*Significance between high-flow nasal cannula and nasal cannula.

### Physiological parameters

Table 4 presents the physiological parameters of the participants. No significant change was observed in most of the physiological parameters, namely CO_2_ (mm Hg) (*p* = 0.72), BT (°C) (*p* = 0.27), PR (per minute) (*p* = 0.40), RR (per minute) (*p* = 0.14), systolic BP (mm Hg) (*p* = 0.74), and diastolic BP (mm Hg) (*p* = 0.93), but a significant change in SpO_2_ (%) was observed (*p* = 0.01).

**Table 4:** The effect of HFNC and NC intervention on physiological parameters.

	HFNC (n = 19)	Mean difference	NC (n = 19)	Mean difference	*p* value
**CO**_**2**_** (mmHg)**					
Before	38.95 ± 4.28	+ 0.11	39.44 ± 4.18	+ 0.80	0.34
After	39.06 ± 3.90		40.24 ± 4.21		
**SpO**_**2**_** (%)**					
Before	96.47 ± 2.32	+ 0.16	96.58 ± 1.84	+ 1.79	0.01*
After	96.63 ± 2.41		98.37 ± 1.54		
**Body temperature (**^**o**^**C)**					
Before	36.43 ± 0.65	+ 0.18	36.52 ± 0.41	+ 0.01	0.27
After	36.61 ± 0.50		36.53 ± 0.34		
**Pulse rate (per minute)**					
Before	87.79 ± 16.02	−7.90	83.79 ± 15.52	−4.53	0.40
After	79.89 ± 13.58		79.26 ± 17.12		
**Respiratory rate (per minute)**					
Before	20.79 ± 2.78	−0.90	21.05 ± 3.36	−1.94	0.14
After	19.89 ± 2.60		19.11 ± 2.08		
**Systolic blood pressure (mmHg)**					
Before	131.00 ± 17.47	−11.0	131.53 ± 21.40	−9.90	0.74
After	120.00 ± 15.53		121.63 ± 15.72		
**Diastolic blood pressure (mmHg)**					
Before	74.79 ± 12.74	−3.16	75.37 ± 13.96	−3.21	0.93
After	71.63 ± 11.50		72.16 ± 11.23		

Analysis of covariance (ANCOVA) test, p < 0.05.

*HFNC* high flow nasal cannula, *NC* nasal cannula, *CO*_*2*_ carbon dioxide, *SpO*_*2*_ peripheral capillary oxygen saturation.

*Significance between high-flow nasal cannula and nasal cannula.

## Discussion

The HFNC treatment significantly improved the R5, R5% predicted, R5–R20, X5-predicted, and Fres IOS values compared with the NC treatment (Table 2). R5 represents the total airway resistance and R5 − R20 reflects resistance in the small airways^39^. Briefly, HFNC significantly reduces total airway resistance (R5) mainly by reducing small airway resistance (R5-R20), compared with NC. HFNC has been shown to improve oxygenation, generates positive airway pressure, increases the end-inspiratory lung volume, reduces inspiratory resistance and the metabolic work associated with gas conditioning, washes out nasopharyngeal dead space, and increases functional residual capacity^20,21,24,25,40–44^ and upper airway opening (e.g., laryngeal opening)^45^. These effects could lead to the reduction of total and mainly small airway resistance shown in our study. Although cold gas potentially induces bronchospasm in patients receiving oxygen therapy via nasal canula. In our study, we demonstrated that the advantages of HFNC in improving respiratory impedance compared with NC not mainly originated from airway spasms induced by cold gas in NC. Other possible mechanisms contributing to small airway resistance reduction may be bronchodilation due to warm and humidified air and a positive end-expiratory pressure effect.

Use of Impulse oscillometry (IOS) is a good approach for measuring both small and large airways resistance and resonance capacitance of the lung in COPD patients. On IOS, respiratory reactance (Xrs) of the lung measures inertance and the elastic properties or compliance of lung periphery. In COPD, X5 reflects the elastic recoil of the peripheral lung tissues and ventilation inhomogeneity due to small airway disease and emphysema^46^. Previous study indicates that while HFNC treatment shows no difference between resistance at 5 Hz and 20 Hz (X5) in healthy individuals, it significantly reduces X5 and Fres in COPD patients. These results indicate use of HFNC may decrease endogenous PEEP, leading to reduction of expiratory resistance^47^. Improvement of hyperinflation or periphery obstruction in lung may be the underlying mechanism. Consistent with previous research findings, our results showed in COPD patients, the beneficial effects of HFNC treatment in improving of X5 and Fres were observed under IOS measurement, which may be due to improvement in small airway obstruction. These were associated with generation of positive airway pressure, increasing the end-inspiratory lung volume, reducing inspiratory resistance, increasing functional residual capacity, bronchodilation due to warm and humidified air, and a positive end-expiratory pressure effect.

IOS parameters have been shown to be more sensitive to bronchial provocation and bronchodilation tests than the traditional PFT^48–50^. IOS has also been recommended to assess bronchodilator pharmacology in patients with COPD^51^, and IOS parameters can be used to accurately differentiate between the effects of pharmacology therapy in cases of similar FEV1 measurements^31^. IOS parameters are highly correlated with traditional pulmonary function parameters and can be used as an alternative to pulmonary function assessments for patients with COPD^52^. The forced oscillation technique is sensitive to bronchodilator effects in patients with airflow obstruction, and these techniques are more sensitive than FEV1 in assessing short-acting bronchodilator effects in patients with COPD^53,54^.

Previous study showed that after bronchodilator administration (2 puffs of albuterol), the IOS parameters such as R5 (from 0.49 to 0.43 kPa/1/s) and R5–20 (from 0.41 to 0.37 kPa/1/s) have significant changed in COPD patients^55^. Accordingly, results from the present study also showed statistical significance of R5 (from 0.58 to 0.54 kPa/L/s) and X5 (from −0.30 to −0.24 kPa/L/s) changes after HFNC treatment for 10 min. While the baseline of R5 values between these two studies seemed comparable and effects of HFNC in IOS parameters did not seem to be exceed the effect of bronchodilators; however, direct efficacy comparison might not be practical, due to different populations and different changes of significant IOS parameters. More studies in efficacy comparison of IOS between bronchodilator and HFNC in COPD patients are warranted. The results of this study indicated that HFNC did not significantly change the PFT parameters of the participants (Table 3), whereas the IOS parameters indicated a significant reduction in small airway resistance and peripheral lung reactance from HFNC (Table 2). This indicates that IOS is more sensitive to detect the decrease in small airway resistance and peripheral lung reactance than the conventional PFT. However, the key practical advantage of IOS is that it uses tidal breathing, which eliminates the possibility of bronchoconstriction during forced expiration in patients with airflow obstruction^56^. This may explain how IOS is more sensitive to detect the changes in resistance in small airways and peripheral lung reactance than the PFT.

Nagata et al. reported that 6-week domiciliary nocturnal HFNC use improved symptoms and quality of life (QOL) and decreased hypercapnia levels in patients with hypercapnia and stable COPD^57^. This may be because heated and humidified gas and mild positive airway pressure have a salutary effect on QOL and the flushing of anatomical dead space and positively affect airway pressure. However, HFNC did not increase pulmonary function, exercise capacity, or physical activity; this suggests that HFNC increases QOL through mechanisms independent of lung mechanics and exercise capacity. In our study, the decrease in small airway resistance and peripheral lung reactance detected through IOS may explain the ability of long-term HFNC use to increase QOL.

For nonpharmacological treatment of COPD, pulmonary rehabilitation plays a vital role because it increases QOL and exercise capacity. Pulmonary rehabilitation is applicable to each level of COPD severity, especially moderate to severe COPD. The meta-analysis review conducted by Mccarthy et al. in 2015 analysed the impact of health-related QOL between pulmonary rehabilitation and usual care and revealed that pulmonary rehabilitation could reduce difficulty breathing and fatigue and improve the emotional function and self-control of patients with COPD^17^. Oxygen therapy can be administered if desaturation occurs during pulmonary rehabilitation. In 2016, Cirio et al. demonstrated that HFNC may improve the performance with an increase around 109 s of endurance time (total around 9 min) of patients with COPD in high-intensity constant-load exercise^58^, and indicated that HFNC potential generated effect 10 min after administration. Therefore, in this study, we designed 10-min HFNC use for evaluation of change of respiratory impedance in COPD patients. But the details of the mechanism remain unclear. Our finding that HFNC decreased small airway resistance and peripheral lung reactance may explain the underlying mechanism. Subsequent randomised control studies should be conducted to evaluate the clinical benefits of long-term pulmonary rehabilitation with HFNC support for patients with stable COPD.

No definitive guidelines for HFNC in patients with stable COPD without chronic respiratory failure have been established. Although our sample size was small, the preliminary data obtained through IOS measurement rather than spirometry or body plethysmography indicated that HFNC improved small airway resistance and peripheral lung reactance compared with NC. The potential of HFNC to decrease small airway resistance and peripheral lung reactance identified in this study may provide reference to develop a new nonpharmacological treatment strategy for patients with stable COPD. However, the effect of respiratory impedance improvement of HFNC may be transient after removing HFNC physiologically. Therefore, breathing difficulty may be further improved after long-term HFNC. Further clinical studies may focus on application of HFNC in COPD patients in a long-term, domiciliary, or rehabilitory manner. Furthermore, clinical impact of HFNC with ambient air for stable COPD patients without respiratory failure may be evaluated. No significant difference in TcPCO_2_ was observed between the HFNC and NC treatments. This may be because our cohort did not contain patients with hypercapnic respiratory failure and because long-term HFNC was not administered. No significant changes in physiological parameters were observed, except for an increase in SpO_2_ with the NC treatment. However, the increase in SpO_2_ from 96 to 98% did not exhibit clinical benefits for patients with stable COPD (Table 4).

The limitations of this study are as follows: (1) the number of patients was relatively small, (2) crossover trials cannot provide an analysis of long-term prognosis, (3) this study was not double blind, (4) we did not measure airway pressure or minute ventilation, which may explain some of the mechanisms and serial effects of the airway, (5) this was a per-protocol analysis, and selection bias may have occurred because of the exclusion or dropping out or patients, (6) the references values for IOS parameters used in this study were adapted from studies on non-Asian populations, (7) the clinical relevance of the difference in IOS parameters was unclear, and (8) the clinical efficacy of different timings and durations, especially long-term usage of HFNC remain unknown, and (9) we cannot compare the efficacy of improvement of respiratory impedance between HFNC and bronchodilator administration because that the usual medications were not discontinued in every participant and each visit, and we did not perform the measurement of IOS before and after bronchodilator. However, this is the first study to evaluate the efficacy of HFNC in improving respiratory impedance through IOS compared with that of NC. A well-designed noncrossover prospective study with a large sample size, clinical outcome evaluation, and comprehensive respirometer measurements or lab examination of body fluids (e.g., blood and urine) should be conducted to further elucidate the impact of HFNC treatment on patients with stable COPD.

## Conclusion

HFNC improved the IOS indicators of small airway resistance and peripheral lung reactance in patients with stable COPD, namely R5, R5% predicted, R5–R20, X5-predicted, and Fres, compared with NC. This is the first study to evaluate the efficacy of HFNC in improving respiratory impedance through IOS compared with that of NC.

## Data Availability

The datasets generated during and/or analyzed during the current study are available from the corresponding author on reasonable request.
